# Live Attenuated Varicella Vaccine: Prevention of Varicella and of Zoster

**DOI:** 10.1093/infdis/jiaa573

**Published:** 2021-09-30

**Authors:** Anne A Gershon, Michael D Gershon, Eugene D Shapiro

**Affiliations:** 1 Columbia University Vagelos College of Physicians and Surgeons, New York, New York, USA; 2 Yale School of Medicine, New Haven, Connecticut, USA

**Keywords:** varicella zoster virus, leukemia, anti-viral therapy, fluorescent antibody to membrane antigen (FAMA), latency, reactivation

## Abstract

Michiaki Takahashi developed the live attenuated varicella vaccine in 1974 . This was the first, and is still the only, herpesvirus vaccine. Early studies showed promise, but the vaccine was rigorously tested on immunosuppressed patients because of their high risk of fatal varicella; vaccination proved to be lifesaving. Subsequently, the vaccine was found to be safe and effective in healthy children. Eventually, varicella vaccine became a component of measles mumps rubella vaccine, 2 doses of which are administered in the USA to ~90% of children. The incidence of varicella has dropped dramatically in the USA since vaccine-licensure in 1995. Varicella vaccine is also associated with a decreased incidence of zoster and is protective for susceptible adults. Today, immunocompromised individuals are protected against varicella due to vaccine-induced herd immunity. Latent infection with varicella zoster virus occurs after vaccination; however, the vaccine strain is impaired for its ability to reactivate.

## HISTORY AND DEVELOPMENT OF THE LIVE ATTENUATED VARICELLA VIRUS VACCINE

Not very long ago, varicella was considered to be a disease of little consequence. Even its popular name, chickenpox, was suggestive of a lack of seriousness. That misconception, however, changed rapidly in the late 1960s and early 1970s, when systemic steroid therapy began more frequently to be administered, organ transplantation was introduced, and childhood cancer started to be cured. The immunosuppression associated with these innovations unmasked the potentially lethal potential of varicella-zoster virus (VZV) and made clear that this seemingly benign virus was a serious pathogen and not just a childhood nuisance. The increasingly serious *Staphylococcus aureus* and group A β-hemolytic streptococcal cellulitis, cerebellitis, and other encephalopathies that complicate primary varicella reinforced that conclusion.

It is now clear that VZV is a herpesvirus, not a poxvirus, although that was not appreciated before the 1950s. Other human herpesviruses include herpes simplex viruses (HSV) 1 and 2, cytomegalovirus (CMV), Epstein-Barr virus (EBV), and herpesviruses 6, 7, and 8. These viruses share the unique ability to cause latent infection in various organs in newly infected hosts. Latent herpesviruses then remain with the individual throughout their lifetime, often without causing symptoms. The site of latent infections varies among the herpesviruses; VZV establishes latency in neurons of peripheral ganglia, as does HSV. Months to years after varicella resolves, VZV may reactivate from latency and cause zoster. Typically, zoster appears as a painful or pruritic unilateral vesicular rash in a dermatomal distribution. Roughly 30% of individuals are thought to develop zoster months to years after having clinical varicella; zoster often occurs in aged or in immunocompromised individuals in whom it may also be severe and become disseminated. Recently, zoster has been reported to be associated with strokes and with diseases involving the gastrointestinal tract such as stomach ulcers (enteric zoster), often occurring without rash [1].

A number of live vaccines for protection of healthy children from viral diseases were successfully introduced before the varicella vaccine. These include poliomyelitis, measles, rubella, and mumps vaccines. For many years, varicella was considered to be too mild to warrant prevention. Serious complications of varicella, such as pneumonia and bacterial sepsis, both of which are now well recognized and justly feared, must have occurred in some children, but may have been ignored. In the 1960s, however, the standard of care for young children with leukemia, which previously had been virtually uniformly fatal, became treatment with anticancer drugs that ameliorated and often cured leukemia, which also were immunosuppressive. In the absence of a vaccine, epidemics of varicella recurred every winter and spring. These epidemics affected susceptible children regardless of whether they were immunosuppressed, but varicella is a dangerous illness in immunosuppressed patient being treated for leukemia. The unanticipated consequence of the advance of medical therapy was that children could be cured of leukemia only to die of varicella. Varicella thus no longer seemed too inconsequential to justify the development of a vaccine. Even in immunocompetent populations, varicella caused approximately 100 deaths/year in the United States. This may not seem like a large number of people but both to the families involved and to society, it was intolerable.

In the 1960s, to address treatment of severe varicella in immunocompromised children, passive immunization, consisting of injection of preformed specific antibodies to VZV, was tried in the United States. Regular immune globulin (IG) modified the risk and severity of varicella if administered soon after a close exposure to VZV, but impractically large volumes of IG were required [2]. Subsequently, IG derived from patients with high titers of antibodies to VZV, specifically those recovering from zoster, was found to be both more efficacious and more practical than IG because smaller volumes were effective [3]. It was later determined that this preparation, termed varicella zoster immune globulin (VZIG), could protect immunocompromised children from severe varicella following exposure to VZV [4]. When administered to immunocompromised patients within a day after exposure, VZIG usually offered protection from severe varicella. The problem with the use of VZIG, however, was that the exposure to VZV had to be recognized; moreover, VZIG was often not available because it was in short supply and was released only after a cumbersome plea to the Centers for Disease Control and Prevention (CDC). Consequently, many children could not receive VZIG even when it was known to be necessary. Although antiviral therapy with acyclovir was becoming available, treatment with acyclovir was not always effective in saving the lives of immunocompromised patients with severe varicella [5]. Because more and more immunocompromised children continued to die from varicella, it became clear that a vaccine was needed.

### Attenuation of VZV to Develop a Vaccine

During the 1960s, scientists in Japan were taking a different approach to preventing severe varicella. Professor Michiaki Takahashi at Osaka University successfully attenuated VZV in his laboratory and produced a live vaccine that could be safely administered to patients who were susceptible to varicella. To attenuate VZV, Takahashi utilized a standard method to diminish viral virulence: serial passages of VZV [6, 7] in cell cultures at varying temperatures. He obtained wild-type VZV from a boy with varicella (his family name was Oka) who was otherwise healthy. Takahashi then passaged the Oka VZV 11 times in human embryonic lung fibroblasts, 12 times in guinea pig fibroblasts, 2 times in WI-38 fibroblasts, and 3–5 times in MRC-5 cells [6]. The multipassaged Oka strain of VZV became the attenuated live viral vaccine (vOka) that appeared to be safe when administered to animals, including primates [8]. However, at the time of Takahashi’s studies, there was no animal model of varicella; therefore, animals could not be used to determine whether vOka actually prevented varicella.

To analyze the safety and efficacy of vOka, the first approach in Japan was to vaccinate small numbers of healthy adults. Further early clinical testing by Takahashi and his colleagues included vaccination of additional healthy adults and children, who did not subsequently develop clinical varicella from the vaccine. These observations strongly suggested that vOka had indeed been attenuated. In addition, many of the vaccinees also appeared to be protected from developing varicella after they were exposed to VZV. Subsequently, children receiving steroids for medical reasons and also some with varying forms of malignancy were immunized to further assess the safety of the vaccine; in some, cancer chemotherapy was withheld for short intervals. The vaccine appeared to stimulate protective immunity; however, protection could not adequately be proven, because it could not be determined whether individuals had protective antibodies to VZV from prior infection before they received the vaccine. In addition, some of the children who were receiving cancer chemotherapy developed rashes after immunization [9–12]. Children with lymphoma seemed especially susceptible to rashes after immunization with vOka. In these early studies, however, the vaccine was shown to be immunogenic, and although definitive proof of the vaccine’s efficacy could not be obtained, vaccinees seemed to be protected from illness. The incidence of zoster in these vaccinees also seemed to be low. Investigations in Japan thus established that vOka had promise as a varicella vaccine but proof of its efficacy required that a test for immunity to VZV be developed. Such a test could be employed to identify truly susceptible individuals prior to vaccination, and used again to evaluate the quality of the immune response to the vaccine.

### Testing the vOka Strain of VZV in the United States

The development of an immune correlate of protection from varicella set the stage for the successful validation of the use of vOka as a vaccine in the United States. This correlate was an immunofluorescence test. The assay used fluorescently labeled antibodies to human IgG to detect antibodies from patients’ serum to antigens expressed on the plasma membranes of human embryonic fibroblasts infected with VZV. This fluorescent antibody to membrane antigen (FAMA) test is now regarded as the gold standard for the detection of protective antibodies to VZV. The FAMA test was clinically validated and shown to indicate protective immunity to varicella. Patients whose sera was FAMA negative became FAMA positive after an episode of varicella. More to the point, patients whose serum was FAMA negative almost invariably acquired varicella after a close exposure to someone with the illness, and patients who had close exposures to someone with varicella failed to develop the illness if their serum was FAMA positive at the time of the exposure [13].

The pioneering studies on development of a live attenuated vaccine against varicella carried out in Japan served as the basis to advance further testing of the safety and, more importantly, of the efficacy of vOka in the United States. The availability of the FAMA test provided a critical advantage for American investigators. There was a fear of vaccinating with a live DNA agent that established latency; however, American studies were undertaken after numerous discussions and meetings of expert virologists, including Professor Takahashi, alleviated this fear. The largest American investigation, which began in 1979, was the Varicella Vaccine Collaborative Study sponsored by the National Institute of Allergy and Infectious Diseases. In this study, 575 children with leukemia that had been in remission for at least 1 year were immunized with 2 doses of vOka given 3 months apart. The leukemic children were still receiving maintenance chemotherapy but this was withheld for 2 weeks after immunization to allow an immune response to occur with less immunosuppression from the chemotherapeutic agents. To participate, the children in the study were required to have varicella-susceptible siblings. Household exposure to VZV was associated with an extremely high risk of infection; therefore, when a sibling developed varicella, the immunized individual was essentially receiving a VZV challenge, providing a rigorous test of protective immunity from the vaccinee. Antibody titers to VZV in the blood of vaccinees were monitored by the FAMA method. Following immunization with 2 doses of vaccine, all of the leukemic children developed FAMA titers that were associated with immunity to varicella. Three per cent developed a troublesome rash from the vaccine itself and required brief treatment with the oral antiviral drug acyclovir for the vaccine-associated rash. If and when their siblings developed varicella, the immunized children were observed closely for any sign of varicella and were not passively immunized with VZIG. If any of the children had acquired varicella, they would have been treated with acyclovir. Of 123 vaccinees whose siblings developed varicella, 106 (86%) did not develop any manifestations of varicella. The other 17 children had very mild infections. None of the exposed children required treatment with acyclovir for varicella rash. Based on historical controls following household exposure, about 80 of these children would have been expected to develop clinical varicella, with a mortality rate approaching 10%. Varicella vaccine was thus shown to be highly protective for children with leukemia in remission [13].

A major concern in the 1970s about use of the newly developed vOka was that it probably caused latent infection. There was some fear that the vaccine virus might reactivate and cause zoster, possibly with a high frequency. Early studies of children with leukemia, however, failed to find the hypothetical increase in zoster; instead, they demonstrated that the frequency of zoster was reduced [14, 15]. In fact, as will be discussed in more detail below, long-term observations have suggested that latency of the vaccine virus may actually confer an advantage, in that periodic asymptomatic reactivation may occur and boost immunity to varicella.

### Early Studies on Immunization of Healthy Children in the United States

Studies of immunocompromised children immunized against varicella in Japan and in the United States indicated that these children benefitted from the vaccine. However, it was feared that if the vaccine was to be given to large numbers of immunocompromised children, it might be difficult to duplicate the complicated conditions in which these studies were conducted, which involved withholding therapy around the time of immunization, frequent monitoring of antibody titers, and careful monitoring exposures to patients with VZV [16]. Consequently, it was generally agreed that it made more practical sense to immunize immunocompetent children routinely against varicella and to utilize the resulting herd immunity to protect immunocompromised children from varicella as was also being done for measles. This decision helped to motivate studies of both the safety and the efficacy of vOka in healthy children, which followed the successful trials of the live attenuated varicella vaccine in leukemic children in the United States. Knowing that the vaccine was safe and effective, even in high-risk immunocompromised children, made it relatively easy to carry out studies in healthy children [16, 17].

In addition to the severity of varicella when it occurs in immunocompromised children, the consequences of varicella can be serious, even when it occurs in otherwise healthy individuals. Varicella is a benign infection except when it is not. The desire to prevent the serious complications of varicella, though they might be uncommon, contributed to acceptance of vOka. These complications include severe and sometimes fatal varicella in adults who did not develop varicella in childhood [18]. This problem is especially common among persons who immigrate to countries in the temperate zone from tropical countries where VZV spreads poorly, making varicella uncommon during childhood [19]. Children appear to have stronger immune responses to VZV than adults, which may contribute to the severity of varicella among adults. In addition, both children and adults with varicella may suffer complications such as encephalitis, meningitis, and severe secondary streptococcal infections [20]. Varicella in pregnant women may also be severe, and newborn infants who develop varicella may have fatal infections. In addition, infants born to women who have varicella during pregnancy may develop the congenital varicella syndrome, which is associated with extensive neurological abnormalities, including mental retardation, blindness, deafness, and abnormal development of the arms and/or legs [15, 19, 20]. It thus became clear that varicella was an infection worth preventing in healthy individuals.

### Differences Between vOka and Wild-Type VZV

In the early analyses of the clinical efficacy of varicella vaccine and its possible complications, it was clear that there needed to be a means of distinguishing between vOka and wild-type VZV (Table 1). The VZV genome comprises 71 genes or open reading frames (ORFs). There are a number of mutations in vOka that are not present wild-type VZV; many are found in ORF 62. In ORF 62, there are 4 consistent single nucleotide point mutations in vOka that distinguish it from wild-type VZV. These mutations are found at positions 105 705, 160 262, 107 252, and 10 811 [15, 21]. It is generally accepted that if a vaccinee develops a severe rash, adverse neurologic events, or other recognized complications of VZV that are temporally or otherwise related to immunization, that the cause of the problem be identified. Close temporal association of an adverse event with administration of vOka does not by itself establish that vOka is the cause of that event. Infection with wild-type VZV or even another virus, such as HSV have to be ruled out. Laboratory testing to identify the cause is indicated. Specimens (such as vesicular fluid, skin swabs, or cerebrospinal fluid [CSF]) are collected and tested to determine whether VZV is present and if so, whether its molecular profile is that of vOka or wild-type VZV [19].

**Table 1.** Differences Between Wild-Type VZV and vOka

Property	Wild-Type VZV	vOka
Pathogenicity	High	Significantly reduced
Transmissibility	Extremely high	Significantly reduced
Severity of infection	Mild to severe	Subclinical to mild
Incidence of zoster after primary infection	Estimated to be 30% over the course of a lifetime	Significantly reduced
RFLP	Distinguishable from vOka	Distinguishable from wild type
Temperature sensitivity in culture	Grows well at 39°C	Temperature sensitive, grows poorly at 39°C
Quantity of gC	Greater than vOka	Less than wild type

Abbreviations: RFLP, restriction fraction length polymorphisms; vOka, Oka strain attenuated live viral vaccine; VZV, varicella-zoster virus.

Exactly which of the many mutations (as many as 42) in vOka are responsible for its attenuation remains unknown. Possibly because many mutations occurred during attenuation, vOka has not been reported to revert to wild type either clinically or structurally. It is also important that vOka was not cloned originally; therefore, the vaccine that is administered is actually composed of a mixture of vOka and wild-type VZVs from the original inoculum. Although recombinant VZVs have been recognized (combinations of vOka and wild-type VZV) to occur on occasion, this is unusual and has not resulted in any recognized clinically virulent strains of virus [15, 20, 21].

### Immune Responses to VZV

Immune responses to VZV after either varicella or immunization include development of both antibodies and cell-mediated immune (CMI) responses [15]. Measurement of antibody titers in clinical laboratories require small amounts of blood and can be automated. Enzyme-linked immunosorbent assay (ELISA) tests are most commonly utilized but have poor sensitivity and are not as accurate as the FAMA test (which has not yet been automated) for assessing protective immunity to varicella after vaccination [15]. The presence of antibodies in blood may play some role in preventing second attacks of varicella, although patients with agammaglobulinemia do not usually have second episodes of this disease. Antibodies, however, do not seem to play a role in recovery from VZV infection. [16, 21]. Antibodies tend, nevertheless, to persist for the lifetime of an individual and are usually present in the blood when patients develop zoster [15, 20]. VZV is highly cell associated; antibodies cannot penetrate the infected cells and destroy the virions. Cellular immunity, which is directed against VZV-infected cells, is crucial for recovery from varicella and also for prevention of and recovery from zoster.

CMI to VZV is mediated by T lymphocytes and natural killer (NK) cells. Testing for CMI to VZV is very complicated. Lymphocytes are needed so large amounts of blood are required [15, 21]. Many complicated steps are involved, the reagents are expensive, and the procedure takes several days, so this testing can only be performed in research laboratories. CMI to VZV decreases with age, especially after age 50; this decline is thought to be the reason that older individuals (in addition to immunocompromised patients) have a high incidence of zoster. Increasing CMI to VZV is the goal of zoster vaccines. Protective levels of CMI required to prevent zoster have not been determined [21, 22].

### Prelicensure Studies of Safety and Efficacy of vOka for Prevention of Varicella in Healthy Children

To determine whether varicella vaccine was effective in healthy children, numerous clinical trials of varicella vaccine were carried out in the United States in the 1980s [15]. Hundreds of healthy children were enrolled. These studies showed that vOka is safe, well tolerated, and protective against household exposures [15]. Side effects were minor and occurred in <5% of vaccinees. Adverse reactions included pain, redness, and rash at the injection site, as well as systemic symptoms of mild generalized rash, and transient fever. Adolescents tended to have more adverse events than healthy younger children. Most immunized children developed ELISA antibodies to VZV. These antibodies persisted for years after vaccination, although whether they are protective is unknown. The vaccine’s safety and immunogenicity were similar in studies of children in both Asia and Europe, where somewhat different formulations of vOka were tested [15, 23, 24].

Overall, roughly 85% of the children who received 1 dose of vOka were protected from wild-type VZV infection after they were exposed to individuals with VZV infections. The few vaccinees who developed varicella after exposure to VZV usually had mild infections. Two double-blind, placebo-controlled studies using different doses of vaccine carried out in the United States [23] and in Finland [24] found that vOka protected 90%–100% of vaccinees over a period of several years. The actual degree of protection depended on both the quantity of attenuated virus in the vaccine that was administered and the intensity of exposure to varicella. Because it is so highly cell associated, VZV is very difficult to propagate. Culturing vOka in quantities adequate to support universal vaccination, and maintaining quality while doing so, thus presented manufacturers with a major challenge that took considerable time to overcome. Small studies in healthy adults who were susceptible to varicella showed that vOka was both safe and effective [25]. In children, higher concentrations of attenuated virus in vOka was associated with higher efficacy against varicella [23, 24]. Live attenuated varicella vaccine was licensed for use in the United States for both children and adults in March 1995 [25]. Licensure of other preparations of vOka in Europe soon followed, with subsequent licensure in a number of countries in Asia, including Japan. All of the currently available vaccines contain the attenuated Oka strain with the exception of one from South Korea, which was attenuated and developed there. Prior to licensure, studies in the United States suggested that a single dose of the vaccine would be cost effective [26, 27]. The cost-effectiveness calculation probably was the critical factor that motivated acceptance of the live attenuated varicella vaccine for licensure.

### Diagnosis of VZV Infections

During the time vOka was being tested, it became possible to use the polymerase chain reaction (PCR) to identify DNA encoding VZV genes in skin lesions [15]. PCR is a much more sensitive and reliable means of identifying VZV than culture of the virus. It is also possible to use PCR and DNA sequencing to distinguish wild-type VZV from vOka in lesions [15]. The availability and reliability of these assays enabled activity of vOka to be accurately analyzed in immunized individuals, enabling reactions to the vaccine to be distinguished from vaccine failures.

### Currently Licensed Vaccines Against Varicella

Vaccines employing vOka have now been licensed around the world, including, Varivax (Merck), Varilrix (Glaxo Smith Kline), and Okavax (Biken). In addition, 4 different Oka vaccines are used in China. SuduVax is used in South Korea and is the only non-Oka strain vaccine. Amounts of VZV virus vary slightly in the different vaccines. Merck and Glaxo Smith Kline market a combination vaccine containing measles, mumps, rubella, and varicella vaccines (MMRV) for use in children at the ages of 12 months to 12 years. Because the measles component is immunosuppressive, a compensatory increase in the amount of vOka had to be used in MMRV. Although the change increased cost, it was worthwhile because the quadrivalent product greatly enhances convenience and thus vaccine uptake. Immunization of immunocompromised patients with vOka is not recommended. As with measles virus, protection from varicella in immunocompromised individuals mainly depends upon herd immunity, requiring widespread vaccine uptake [15].

According to the CDC, evidence of immunity to varicella includes any of the following: documented age-appropriate vaccination (1 or 2 doses depending on the age of the child), birth in the United States before 1980, or verified history of varicella or zoster. (Pregnant women, healthcare personnel, and immunocompromised individuals require a more thorough evaluation than the general population.) Measurement of levels of antibodies to VZV after immunization of children or adults adds to the expense of vaccination and does not necessarily provide useful information.

### Postlicensure Experience With Live Attenuated Varicella Vaccine

After vOka was licensed in the United States, the vaccine continues to be evaluated. Immunized children and adults who manifest a serious rash or other symptoms post vaccination are identified and studied. Postlicensure safety studies are mandated by the Food and Drug Administration because they provide information on safety and efficacy. The Worldwide Adverse Experience System sponsored by Merck was established in the late 1990s. It analyzes potential adverse events following VZV vaccines. This program seeks to determine whether VZV recovered from patients with an adverse event after receiving the vaccine is vOka or wild-type VZV. The CDC also performs such laboratory determinations [15].

The type of VZV present in a rash that occurs shortly after vaccination continues to be of interest. The vOka in varicella vaccine is a live agent and could, in theory, infect an individual and give rise to disease despite its attenuation. The vaccine could occasionally be administered to an individual who is incubating wild-type VZV. After vaccination, a rash caused by infection with VZV could either be due to an adverse effect of vOka or it could be due to infection with wild-type VZV. If the latter, the rash could be the result of infection that occurred prior to vaccination or it might indicate vaccine failure. During the early days after vOka was licensed, this type of analysis was particularly important because there were still many children who actually were developing varicella. At that time, it was more common to identify wild-type VZV than vOka in postvaccine rashes. After vaccination became widespread and relatively little wild-type VZV was circulating, the presence of wild-type VZV in a rash most often occurred as a result of zoster; that is, VZV reactivated in a peripheral ganglion that extended to and secondarily infected the skin. Studies indicated that zoster could be caused either by vOka or by wild-type VZV. Despite vaccination with varicella vaccine, there may have been either previous or subsequent subclinical infection by wild-type VZV, with associated latent infection with wild-type VZV that can reactivate and give rise to zoster [15].

## IMPACT OF LIVE ATTENUATED VARICELLA VACCINE

In addition to relatively short-term efficacy, it is important to determine the duration of protective immunity and other aspects of impact that the vaccine confers against VZV.

### Impact of 1 Dose

In the United States, varicella vaccine was licensed in 1995 for routine immunization of healthy susceptible children and adults. Within 10 years of licensure, a time when only 1 dose was recommended for children <13 years of age, 88% of American children had been immunized [16]. Because varicella was not a reportable disease then, it was not possible to assess the impact of the vaccine in the entire country. In 1995, the CDC established an active varicella surveillance program in sites in California, Pennsylvania, and Texas with a combined population of 1.2 million. The surveillance programs in California and Pennsylvania were maintained for 15 years. During this period, reported cases of varicella in these areas declined by over 70% while hospitalizations declined by 80%. By 2005, more than 90% of the target population had received the vaccine [15]. The numbers and severity of school and childcare outbreaks decreased significantly. Importantly the death rate from varicella was significantly diminished by 97% in children, adolescents, and adults. There was no evidence of an increase in varicella in older age groups as had once been feared [15]. The vaccine’s effectiveness remained substantial up to 8 years after vaccination in a case-control study [28].

### Impact of 2 Doses

**Figure 1.**
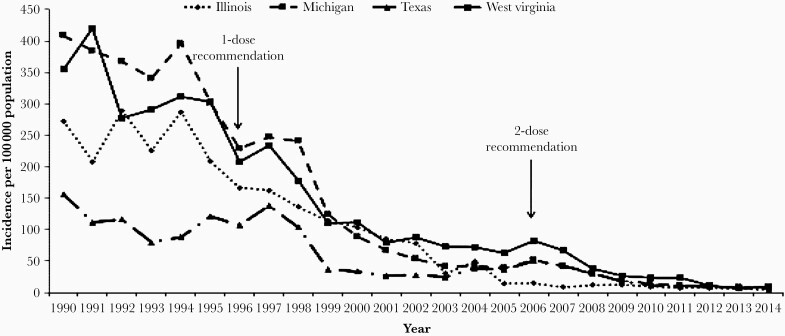
Centers for Disease Control and Prevention reported incidence of varicella in 4 representative states 1990–2014. Varicella-zoster virus was attenuated and vOka was reported as a potential live attenuated vaccine in 1974, testing began in immunocompromised children in 1979, the efficacy and safety of the live attenuated varicella vaccine in immunocompromised and healthy children were reported in 1983–1984, and the vOka-based vaccine was finally licensed in the United States in 1995. The efficacy of the vaccine and the 2006 recommendation for a second dose are illustrated in the figure.

Although between 1995 and 2006, 1 dose of varicella vaccine was routinely administered to most children; it became apparent that frequent outbreaks of varicella continued to occur in many schools even though most students had been vaccinated (Figure 1). Although varicella was mild in most affected children, some developed severe varicella, and the outbreaks also were forcing schools to close for a period of time. The problem of school outbreaks went on for some time, as there was reluctance to introduce a 2-dose schedule of varicella vaccine for children to try to rectify the problem. These frustrating outbreaks were expensive to deal with in families in which both parents were employed and they also interfered with effective schooling. Two doses of varicella vaccine given during childhood had proved to be highly successful in preventing varicella in both adults and immunocompromised patients [15]. A study of vaccinated children from several different locations using the FAMA assay indicated that after a single dose of vaccine a substantial proportion of vaccinees (24%) failed to develop concentrations of antibodies associated with protection from VZV [29]. Another case-control study of children found that 2 doses of vOka provided significantly better protection than a single dose [29]. In 2007, after much deliberation, the CDC recommended that a second dose of varicella vaccine be given routinely (Table 2) [30]. That recommendation was widely followed and school outbreaks became less frequent and less problematic. By 2010, over 90% of children in the United States were immunized with 2 doses of vOka, and fewer than 100 cases of varicella were reported to the CDC annually. Hospitalizations for complications of varicella were fewer than 50 per year, and mortality from varicella in children and adults younger than 50 years of age became extremely rare. There was no evidence of a shift in the incidence of varicella from children to young adults or any other evidence that might suggest that immunity to VZV wanes in vaccinees who had received 2 doses of the vaccine.

**Table 2.** Current Recommendations for Varicella Vaccine in the United States (Centers for Disease Control and Prevention, 2007)

Category of Person Immunized	Routine Childhood Schedule: 2 Doses (at Least 3 mo Apart)	
	First Dose	Second Dose
Preschool and school children	12–15 mo of age	4–6 y of age or before child enters school Not given if child has prior episode of breakthrough varicella
Persons older than 13 y	As soon as conveniently possible	4–8 wk after first dose

### Nonsevere Adverse Events Associated with Varicella Vaccine

After licensure of varicella vaccine, potential adverse events due to vaccination with varicella vaccine continued to be monitored [15, 31]. As expected, the nature and incidence of adverse events after vOka was licensed were generally similar to those observed in prelicensure studies. Adverse events, which occurred in only a small percentage of vaccinees (<5%), were mild. They typically consisted of transient rashes, local reactions at the injection site, or transient low-grade fever. Reactions were less common after the second dose than after the first. vOka was not recommended for immunocompromised children. Rarely, however, vOka was administered to immunodeficient individuals mistakenly who, at the time of vaccination, were thought to be immunocompetent. In at least some of these situations, complications of vOka in the form of vaccine-associated varicella occurred [15]; nevertheless, with some exceptions (see below), these cases were usually not severe and responded well to antiviral chemotherapy.

In 2005, MMRV vaccine was licensed for use in the United States. This was a major advance because it meant that children could receive fewer injections. There was, however, an increase in the frequency of fever and hence a slight increase in the absolute number of febrile seizures in children who received their first dose of MMRV around 12 months of age. This led the CDC to announce a preference for separate administrations of MMR and the monovalent varicella vaccine for the initial doses. This decision became highly controversial and eventually was modified. Because febrile seizures rarely have adverse sequelae and also are rare after MMRV, the CDC now recommends that parents be allowed to choose which approach they prefer for their children [15]. The current recommendation is to involve parents in the decision whether to give MMRV or separate injections of MMR and varicella vaccine for their first doses of these vaccines.

Gastrointestinal disease in the form of abdominal pain and gastric ulcers has recently been reported to occur in some vaccinees, usually at a long interval following vaccination [1]. VZV was discovered in 2015 to establish latency in the enteric nervous system. It is thus not surprising that vOka, as well as wild-type VZV, can be latent in neurons within the gut. Cases of vOka-induced gastrointestinal disease, as a form of zoster without rash, have been reported; the frequency of “enteric zoster” due to vOka is not yet known and is currently being studied. These infections have mainly been associated with abdominal pain and appear to be either self-limited or responsive to antiviral therapy.

### Serious Complications Attributed to Varicella Vaccine

Serious complications after receipt of the vOka varicella vaccine are rare. A number of patients with illness with temporal proximity to vaccination, presumed to be due to vOka, were subsequently identified molecularly as caused by wild-type VZV [15]. These complications included: zoster, pneumonia, meningitis, stroke, and thrombocytopenia. In rare instances, serious reactions to vOka have occurred in immunocompromised patients whose immunodeficiency was unrecognized when they were vaccinated. These complications included disseminated infections, pneumonia, hepatitis, and meningitis. Associated immunodeficiencies have included abnormalities in NK cells, human immunodeficiency virus (HIV) infection, and severe combined immunodeficiency [32]. Only rarely have these infections not responded to antiviral therapy. After 25 years of experience in the United States, 6 fatal cases of VZV infection caused by vOka have been described, all in either children or adults with immunodeficiency who were inadvertently vaccinated (2 had received live zoster vaccine) [15, 33–38]. In the prevaccine era, many more deaths due to natural VZV infections were reported in immunocompromised patients.

Special mention should be made regarding VZV meningitis, which may occur at any interval after either varicella or receipt of vOka, and has been described in otherwise healthy and immunocompromised patients. The advent of widely available and accurate molecular diagnostic testing on CSF has led to identification of many cases of VZV meningitis that would previously have gone undiagnosed [39]. Often these patients have no rash, their CSF is concomitantly tested for numerous viruses that cause meningitis in routine viral diagnostic laboratories, and VZV is identified [15, 39]. Whether this is a true increase in VZV meningitis or is improved identification of VZV due to advanced technology is unknown and deserves further study. Rarely, the Oka strain has been identified in the CSF of such patients. Fortunately, the prognosis of treated VZV meningitis due to any strain of VZV in immunocompetent patients is excellent. Most patients have responded favorably to antiviral therapy [15, 39]. Although ischemic stroke is a rare complication of wild-type varicella and zoster, stroke has not been identified to be associated with vOka [15].

### Transmission of vOka to Others

Wild-type VZV, which is highly contagious, is spread from cutaneous vesicles [15]. The primary means of dissemination of VZV from one host to another is the sloughing of infected squames from the skin, which drift though the air for long periods and can be inhaled by a susceptible individual who comes within range. Reports of transmission of vOka from vaccinees to others are extremely rare. Since the licensure of the attenuated live varicella vaccine in 1995, more than 150 million doses have been distributed worldwide. Only 11 instances of transmission to others have been identified [15]. Transmission of vOka requires many cutaneous lesions on the vaccinee; the more vesicles that are present on the skin, the greater the risk of transmission [15]. If no skin lesions are present, transmission of vOka has not been reported [40–42]. Because development of extensive cutaneous vesicles in vaccinees is necessary for vOka to be transmitted to others, it is to be expected that such transmission is uncommon. Only rarely can VZV be cultured from respiratory secretions [43].

### Breakthrough Infections

When individuals who have been immunized develop varicella with wild-type VZV, the illness is termed a breakthrough infection. Such conditions are uncommon and usually are mild. Children with breakthrough infections, however, can transmit wild-type VZV to other individuals who are susceptible to varicella. Persons with breakthrough varicella who develop more than 50 skin vesicles are more likely to transmit wild-type VZV to others than individuals with smaller numbers of vesicles [42, 44].

### Zoster Following Vaccination

When vOka was first developed, it was feared that zoster might become more common after immunization than after infection with wild-type VZV. Although vOka was attenuated and thus did not cause a significant acute illness, little was known at the time about its tendency to develop latency and subsequently to reactivate. Consequently, a great deal of research was carried out to compare the incidence of zoster in immunized individuals with those who had had wild-type varicella. Early studies were conducted in children with leukemia who were immunized in the 1980s. It was found that the incidence of zoster in these vaccinees was significantly lower than that among similar leukemic children who had had varicella from wild-type VZV [14]. Because what happened in leukemic children might not be generalizable to a healthy population, there was also considerable interest in determining the incidence of zoster in healthy individuals who had received vOka. Two large studies that utilized electronic databases were carried out by members of Kaiser Permanente and the CDC. In the first study done between 2005 and 2009, 322 subjects were enrolled, of whom 118 had been vaccinated. The incidence of zoster was 79% lower in vaccinated children than in unvaccinated children [45]. Interestingly, half of the cases of zoster detected in vaccinees were caused by wild-type VZV, suggesting either that they had been asymptomatically infected with wild-type VZV after they received the vaccine or that they had already been infected with wild-type VZV prior to vaccination. A subsequent study involving children vaccinated between 2004 and 2014 found that the incidence of zoster was 72% lower in vaccinated children than in those who had had varicella from wild-type VZV [46]. Evidently, vOka protects against 2 diseases, zoster as well as varicella. The protection against zoster is an unanticipated benefit and needs further study. When zoster due to vOka does develop, however, it does not appear to be milder than that caused by wild-type VZV. Studies have demonstrated that vOka is not attenuated for establishment of latency; vOka does appear, however, to be attenuated with respect to its tendency to reactivate [47].

### Evidence of Attenuation

As would be expected, much of the evidence that vOka is attenuated comes from clinical information about vaccinees. One important piece of evidence is that the natural illness, varicella, in which the average number of cutaneous vesicles is over 100, is much more severe than the mild rashes due to vOka that occur in a small proportion of immunized individuals about a week after vaccination. These vOka rashes may be generalized in distribution, but almost always consist of only a few vesicles. Varicella caused by wild-type VZV almost invariably results in a widespread pruritic rash that evolves over a few days in crops thought to be a consequence of intermittent viremia. VZV is sloughed from the epidermis of cutaneous vesicles and is highly contagious. In contrast, vOka sloughed from the small numbers of vesicles that occasionally occur after vaccination is rarely infectious to others. When there is an extensive rash due to vOka, as may occur if an immunocompromised individual is vaccinated, vOka can be infectious [41]. About 20% of leukemic vaccinees who developed a vaccine-associated rash transmitted a very mild form (often subclinical) of varicella to their siblings. None of the exposed siblings developed full-blown varicella [40]. In contrast, wild-type VZV infection is rarely asymptomatic or subclinical [15]. This is additional strong evidence of the attenuation of vOka. As noted previously (see section “Differences Between vOka and Wild-Type VZV”) there is no evidence that recombination of vOka leads to enhanced virulence.

### Does Widespread Vaccination of Children Increase the Probability that Zoster will Increase in Unvaccinated Adults in the Future?

At the beginning of the 21st century, some hypothesized that the incidence of zoster would increase because circulating VZV and periodic epidemics of varicella were needed to boost immunity to levels sufficient to suppress reactivation of VZV in adults who previously had varicella. Essentially, this idea supposes that in a population it is necessary for children to develop varicella to minimize the occurrence of zoster in adults. The idea is a reversal of what is commonly accepted as ethical, that adults should protect defenseless children. In the case of zoster, defenseless children are expected to endure the risks of a disease (varicella) to shield adults from the discomfort of zoster. It is important to examine this issue because individuals who oppose vaccination could employ this idea as an excuse to stop the use of varicella vaccine in children. In 2002, a report used epidemiologic computer modeling to predict that universal vaccination in the United States would cause an extra 21 million cases of zoster and an extra 5000 deaths to occur within 50 years because of an increase in the incidence of zoster. This analysis was predicated on the assumption that maintenance of immunity to VZV was absolutely dependent on boosts derived from exposure to VZV released from VZV-infected children during annual epidemics of varicella [48]. Because of this report, in some countries, such as England, fear of a consequent increase in zoster precluded use of varicella vaccine for routine immunization of healthy children. It is clear that exposure of a person who is immune to VZV to an individual with varicella who is shedding the virus can boost immunity [49]. The question is whether such boosting of immunity is *required* for suppression of reactivation of VZV and prevention of zoster. Boosting of immunity due to random exposures to children with varicella during epidemics has not been demonstrated to prevent zoster [50]. The incidence of zoster, furthermore, in cloistered nuns and monks who lived in the complete absence of contact with children, was the same as that of a matched control group who lived with children [51]. These observations suggest that exposure to children with varicella is not a critical factor in the maintenance of immunity to VZV. Multiple subclinical reactivations of VZV may occur spontaneously and, despite not causing clinical disease, may still provide an endogenous boost to immunity against zoster. In any case, the current availability of an efficacious vaccine against zoster mitigates this theoretical objection to varicella vaccination.

The incidence of zoster began to increase in the 1950s, long before vOka was developed, and apparent increases in the incidence of zoster has occurred in countries that use varicella vaccine as well as in those that do not [50]. The introduction of universal vaccination has not changed the slope of the increase in the incidence of zoster in the United States [50]. The increase in zoster is most likely multifactorial and related to enhanced diagnosis, as well as parallel increases in the numbers of immunocompromised individuals from diseases and treatments that suppress immunity as adverse effect, and increased numbers of elderly persons [50].

Endogenous boosting of immunity probably does occur, as was first proposed by Hope-Simpson in 1965 [52]. He postulated that VZV established latency following varicella and that it reactivated periodically. Most of these reactivations were asymptomatic and each was hypothesized to boost immunity to VZV. Aging or other unidentified factors, such as time, caused the overall high level of immunity to VZV to sink below a threshold needed to prevent the symptomatic manifestation of clinically apparent zoster. When that happened, clinical zoster occurred, but would also provide a strong boost to immunity. Much of Hope-Simpson’s hypothesis has been verified. Asymptomatic reactivation of VZV was recently proven when 30% of apparently healthy astronauts returning from space travel were found to have had reactivation of VZV, usually without symptoms [53]. PCR was employed to identify the temporary presence of VZV DNA in their saliva. Others have also demonstrated silent reactivation of VZV in both children and adults [16]. Silent reactivation of VZV with continual boosting may be critical for the maintenance of long-term immunity to varicella in vaccinees, and might explain why significant loss of immunity after vOka has not been observed. Such a mechanism for preservation of long-term immunity to varicella could only come from a vaccine that induces a latent infection. This hypothesis has implications for development of future varicella vaccines; an inactivated or a component vaccine that does not establish latency might not be able to induce as long-lasting an immunity to VZV as that produced by vaccination with vOka. On the other hand, neither an inactivated nor a component vaccine would be capable of reactivation to cause zoster. Because vOka has been used for universal vaccination in the United States and in other countries, it will be important to continue to follow vaccinated individuals to be certain that immunity to VZV persists, as it is thought to do, for many years following vaccination.

In conclusion, varicella used to be very common in the United States. In the early 1990s, about 4 million people, mostly children, developed varicella annually. As a result, from 10 500 to 13 000 individuals were hospitalized every year, and 100 to 150 of them died. vOka was licensed for use in the United States in 1995. Since then, the vaccine has prevented more than 3.5 million cases of varicella, 9000 hospitalizations, and 100 deaths each year in the United States. This is a greater than 90% decrease in the morbidity and mortality due to varicella. The use of a single dose of vOka between 2005 and 2006 caused an 85% decline in the incidence of varicella in children. An even greater decrease occurred after the second dose of vOka was instituted. Hospitalizations for varicella had diminished by 93% in 2012. Varicella deaths during 2012–2016 were 94% lower than they were in 1990–1994. In children and adolescents less than 20 years of age, deaths from varicella declined by 99% during similar periods. The use of varicella vaccine has also induced herd immunity that protects individuals who cannot be vaccinated. The incidence of varicella among infants—a group not eligible for the vaccine—has declined by 90% from 1995 to 2008. Similarly, there was a decline in the incidence of varicella among HIV-infected children, who may not be able to be vaccinated. The rate of zoster also declined significantly in vaccinated children and adolescents, probably because vOka is less likely to reactivate than is wild-type VZV.

A number of studies indicate that antibodies to VZV persist for many years after vaccination, as does CMI [15]. There has been no indication of waning immunity after vaccination as was feared when vaccine programs were being developed. There has been no evidence that significant numbers of vaccinated children develop varicella over time. The American experience with universal varicella vaccination is both the longest and the best documented in the world. That experience is a useful model for other countries to follow. It is critical, however, to continue to monitor protection against varicella in vaccinees. The price of liberty from varicella and zoster is eternal vigilance of the duration of immunity to VZV.

## Notes

***Financial support***. This work was supported by the National Institute of Diabetes and Digestive and Kidney Disease (grant number R01 NIDDK093094 to A. A. G., M. D. G., and E. D. S.); and the National Center for Advancing Translational Science (grant number CTSA UL1 TR000142 to E. D. S.).

***Supplement sponsorship.*** This supplement is sponsored by the Bill and Melinda Gates Foundation.

***Potential conflicts of interest.*** All authors: No reported conflicts of interest. All authors have submitted the ICMJE Form for Disclosure of Potential Conflicts of Interest. Conflicts that the editors consider relevant to the content of the manuscript have been disclosed.

## References

[1] Gershon AA, Chen J, Gershon MD. Use of saliva to identify varicella zoster virus infection of the gut. Clin Infect Dis 2015; 61:536–44. 25882301 10.1093/cid/civ320 PMC4607733

[2] Ross AH. Modification of chicken pox in family contacts by administration of gamma globulin. N Engl J Med 1962; 267:369–76. 14494142 10.1056/NEJM196208232670801

[3] Brunell PA, Ross A, Miller LH, Kuo B. Prevention of varicella by zoster immune globulin. N Engl J Med 1969; 280:1191–4. 4181206 10.1056/NEJM196905292802201

[4] Gershon AA, Steinberg S, Brunell PA. Zoster immune globulin. A further assessment. N Engl J Med 1974; 290:243–5. 4358055 10.1056/NEJM197401312900503

[5] Feldman S, Lott L. Varicella in children with cancer: impact of antiviral therapy and prophylaxis. Pediatrics 1987; 80:465–72. 2821476

[6] Takahashi M, Otsuka T, Okuno Y, Asano Y, Yazaki T. Live vaccine used to prevent the spread of varicella in children in hospital. Lancet 1974; 2:1288–90. 4139526 10.1016/s0140-6736(74)90144-5

[7] Takahashi M, Okuno Y, Otsuka T, Osame J, Takamizawa A. Development of a live attenuated varicella vaccine. Biken J 1975; 18:25–33. 167707

[8] Takahashi M, Asano Y, Kamiya H, Baba K. Development of live varicella vaccine. Nihon Rinsho 1985; 43:1535–41. 2997506

[9] Takahashi M, Kamiya H, Baba K, Asano Y, Ozaki T, Horiuchi K. Clinical experience with Oka live varicella vaccine in Japan. Postgrad Med J 1985; 61:61–7. 3014479

[10] Takahashi M, Asano Y, Kamiya H, Baba K. Varicella vaccine: case studies. Microbiol. Sci 1985; 2:249–54. 2856380

[11] Takahashi M. Clinical overview of varicella vaccine: development and early studies. Pediatrics 1986; 78: 736–41. 3020493

[12] Takahashi M. The varicella vaccine. Vaccine development. Infect Dis Clin North Am 1996; 10:469–88. 8856348 10.1016/s0891-5520(05)70309-3

[13] Gershon AA, Steinberg SP, Gelb L, et al. Live attenuated varicella vaccine: efficacy for children with leukemia in remission. JAMA 1984; 252:355–62. 6330386 10.1001/jama.252.3.355

[14] Hardy I, Gershon AA, Steinberg SP, LaRussa P. The incidence of zoster after immunization with live attenuated varicella vaccine. A study in children with leukemia. Varicella Vaccine Collaborative Study Group. N Engl J Med 1991; 325:1545–50. 1658650 10.1056/NEJM199111283252204

[15] Gershon A, Marin M, Seward JF. Live attenuated varicella vaccine. In: Plotkin S, Orenstein WA, Offit PA, Edwards KM, eds. Vaccines. Philadelphia: WB Saunders, 2018:1145–80.

[16] White CJ. Clinical trials of varicella vaccine in healthy children. Infect Dis Clin North Am 1996; 10:595–608. 8856354 10.1016/s0891-5520(05)70315-9

[17] Choo PW, Donahue JG, Manson JE, Platt R. The epidemiology of varicella and its complications. J Infect Dis 1995; 172:706–12. 7658062 10.1093/infdis/172.3.706

[18] Maretic Z, Cooray MP. Comparisons between chickenpox in a tropical and a European country. J Trop Med Hyg 1963; 66:311–5. 14090785

[19] Gershon AA, Breuer J, Cohen JI, et al. Varicella zoster virus infection. Nat Rev Dis Primers 2015; 1:15016. 27188665 10.1038/nrdp.2015.16 PMC5381807

[20] Breuer J. Herpes zoster: new insights provide an important wake-up call for management of nosocomial transmission. J Infect Dis 2008; 197:635–7. 18260760 10.1086/527421

[21] Levin MJ. Zoster vaccine. In: Plotkin S, Orenstein W, Offit PA, Edwards KM, eds. Vaccines. Philadelphia: WB Saunders, 2008:1057–68.

[22] Gershon AA, Gershon MD. Pathogenesis and current approaches to control of varicella-zoster virus infections. Clin Microbiol Rev 2013; 26:728–43. 24092852 10.1128/CMR.00052-13 PMC3811230

[23] Weibel RE, Neff BJ, Kuter BJ, et al. Live attenuated varicella virus vaccine. Efficacy trial in healthy children. N Engl J Med 1984; 310:1409–15. 6325909 10.1056/NEJM198405313102201

[24] Varis T, Vesikari T. Efficacy of high-titer live attenuated varicella vaccine in healthy young children. J Infect Dis 1996; 174(suppl 3):S330–4. 8896541 10.1093/infdis/174.supplement_3.s330

[25] Gershon AA, Steinberg SP, LaRussa P, Ferrara A, Hammerschlag M, Gelb L. Immunization of healthy adults with live attenuated varicella vaccine. J Infect Dis 1988; 158:132–7. 2839577 10.1093/infdis/158.1.132

[26] Preblud SR, Orenstein WA, Koplan JP, Bart KJ, Hinman AR. A benefit-cost analysis of a childhood varicella vaccination programme. Postgrad Med J 1985; 61:17–22. 3939152

[27] Lieu TA, Cochi SL, Black SB, et al. Cost-effectiveness of a routine varicella vaccination program for US children. JAMA 1994; 271:375–81. 8283587

[28] Vázquez M, LaRussa PS, Gershon AA, et al. Effectiveness over time of varicella vaccine. JAMA 2004; 291:851–5. 14970064 10.1001/jama.291.7.851

[29] Shapiro ED, Vazquez M, Esposito D, et al. Effectiveness of 2 doses of varicella vaccine in children. J Infect Dis 2011; 203:312–5. 21208922 10.1093/infdis/jiq052 PMC3071110

[30] Marin M, Güris D, Chaves SS, et al. Prevention of varicella: recommendations of the Advisory Committee on Immunization Practices (ACIP). MMWR Recomm Rep 2007; 56:1–40. 17585291

[31] Willis ED, Woodward M, Brown E, et al. Herpes zoster vaccine live: a 10 year review of post-marketing safety experience. Vaccine 2017; 35:7231–9. 29174682 10.1016/j.vaccine.2017.11.013 PMC5739308

[32] Gershon AA, Gershon MD. Perspectives on vaccines against varicella-zoster virus infections. Curr Top Microbiol Immunol 2010; 342:359–72. 20232192 10.1007/82_2010_12 PMC5391036

[33] Schrauder A, Henke-Gendo C, Seidemann K, et al. Varicella vaccination in a child with acute lymphoblastic leukaemia. Lancet 2007; 369:1232. 17416267 10.1016/S0140-6736(07)60567-4

[34] Bhalla P, Forrest GN, Gershon M, et al. Disseminated, persistent, and fatal infection due to the vaccine strain of varicella-zoster virus in an adult following stem cell transplantation. Clin Infect Dis 2015; 60:1068–74. 25452596 10.1093/cid/ciu970 PMC4447792

[35] Costa E, Buxton J, Brown J, et al. Fatal disseminated varicella zoster infection following zoster vaccination in an immunocompromised patient. BMJ Case Rep 2016; 2016:bcr2015212688. 10.1136/bcr-2015-212688 PMC4885517 27147629

[36] Leung J, Siegel S, Jones JF, et al. Fatal varicella due to the vaccine-strain varicella-zoster virus. Hum Vaccin Immunother 2014; 10:146–9. 23982221 10.4161/hv.26200 PMC4181020

[37] Alexander KE, Tong PL, Macartney K, Beresford R, Sheppeard V, Gupta M. Live zoster vaccination in an immunocompromised patient leading to death secondary to disseminated varicella zoster virus infection. Vaccine 2018; 36:3890–3. 29807711 10.1016/j.vaccine.2018.05.078

[38] Dutmer CM, Asturias EJ, Smith C, et al. Late onset hypomorphic RAG2 deficiency presentation with fatal vaccine-strain VZV infection. J Clin Immunol 2015; 35:754–60. 26515615 10.1007/s10875-015-0207-8 PMC4662621

[39] Harrington WE, Mato S, Burroughs L, et al. Vaccine Oka varicella meningitis in two adolescents. Pediatrics 2019; 144:e20191522. 31776194 10.1542/peds.2019-1522 PMC6889945

[40] Tsolia M, Gershon AA, Steinberg SP, Gelb L, et al. Live attenuated varicella vaccine: evidence that the virus is attenuated and the importance of skin lesions in transmission of varicella-zoster virus. J Pediatr 1990; 116:184–9. 2153790 10.1016/s0022-3476(05)82872-0

[41] Marin M, Leung J, Gershon AA. Transmission of vaccine-strain varicella-zoster virus: a systematic review. Pediatrics 2019; 144:e20191305. 31471448 10.1542/peds.2019-1305 PMC6957073

[42] Seward JF, Zhang JX, Maupin TJ, Mascola L, Jumaan AO. Contagiousness of varicella in vaccinated cases: a household contact study. JAMA 2004; 292:704–8. 15304467 10.1001/jama.292.6.704

[43] Gold E. Serologic and virus-isolation studies of patients with varicella or herpes-zoster infection. N Engl J Med 1966; 274:181–5. 4285450 10.1056/NEJM196601272740403

[44] Leung J, Broder KR, Marin M. Severe varicella in persons vaccinated with varicella vaccine (breakthrough varicella): a systematic literature review. Expert Rev Vaccines 2017; 16:391–400. 28276305 10.1080/14760584.2017.1294069 PMC5544348

[45] Weinmann S, Chun C, Schmid DS, et al. Incidence and clinical characteristics of herpes zoster among children in the varicella vaccine era, 2005-2009. J Infect Dis 2013; 208:1859–68. 23922376 10.1093/infdis/jit405

[46] Weinmann S, Naleway AL, Koppolu P, et al. Incidence of herpes zoster among children: 2003–2014. Pediatrics 2019; 144:e20182917. 31182552 10.1542/peds.2018-2917 PMC7748320

[47] Sadaoka T, Depledge DP, Rajbhandari L, Venkatesan A, Breuer J, Cohen JI. In vitro system using human neurons demonstrates that varicella-zoster vaccine virus is impaired for reactivation, but not latency. Proc Natl Acad Sci U S A 2016; 113:E2403–12. 27078099 10.1073/pnas.1522575113 PMC4855584

[48] Brisson M, Gay NJ, Edmunds WJ, Andrews NJ. Exposure to varicella boosts immunity to herpes-zoster: implications for mass vaccination against chickenpox. Vaccine 2002; 20:2500–7. 12057605 10.1016/s0264-410x(02)00180-9

[49] Gershon AA, LaRussa P, Steinberg S, Mervish N, Lo SH, Meier P. The protective effect of immunologic boosting against zoster: an analysis in leukemic children who were vaccinated against chickenpox. J Infect Dis 1996; 173:450–3. 8568309 10.1093/infdis/173.2.450

[50] Harpaz R. Do varicella vaccination programs change the epidemiology of herpes zoster? A comprehensive review, with focus on the United States. Expert Rev Vaccines 2019; 18:793–811. 31318605 10.1080/14760584.2019.1646129

[51] Gaillat J, Gajdos V, Launay O, et al. Does monastic life predispose to the risk of Saint Anthony’s fire (herpes zoster)? Clin Infect Dis 2011; 53:405–10. 21844022 10.1093/cid/cir436

[52] Hope-Simpson RE. The nature of herpes zoster: a long term study and a new hypothesis. Proc Roy Soc Med 1965; 58:9–20. 14267505 10.1177/003591576505800106 PMC1898279

[53] Mehta SK, Cohrs RJ, Forghani B, Zerbe G, Gilden DH, Pierson DL. Stress-induced subclinical reactivation of varicella zoster virus in astronauts. J Med Virol 2004; 72:174–9. 14635028 10.1002/jmv.10555

